# Reverse network diffusion to remove indirect noise for better inference of gene regulatory networks

**DOI:** 10.1093/bioinformatics/btae435

**Published:** 2024-07-04

**Authors:** Jiating Yu, Jiacheng Leng, Fan Yuan, Duanchen Sun, Ling-Yun Wu

**Affiliations:** School of Mathematics and Statistics, Nanjing University of Information Science & Technology, Nanjing 210044, China; IAM, MADIS, NCMIS, Academy of Mathematics and Systems Science, Chinese Academy of Sciences, Beijing 100190, China; School of Mathematical Sciences, University of Chinese Academy of Sciences, Beijing 100049, China; IAM, MADIS, NCMIS, Academy of Mathematics and Systems Science, Chinese Academy of Sciences, Beijing 100190, China; School of Mathematical Sciences, University of Chinese Academy of Sciences, Beijing 100049, China; Zhejiang Lab, Hangzhou 311121, China; IAM, MADIS, NCMIS, Academy of Mathematics and Systems Science, Chinese Academy of Sciences, Beijing 100190, China; School of Mathematical Sciences, University of Chinese Academy of Sciences, Beijing 100049, China; School of Mathematics, Shandong University, Jinan 250100, China; IAM, MADIS, NCMIS, Academy of Mathematics and Systems Science, Chinese Academy of Sciences, Beijing 100190, China; School of Mathematical Sciences, University of Chinese Academy of Sciences, Beijing 100049, China

## Abstract

**Motivation:**

Gene regulatory networks (GRNs) are vital tools for delineating regulatory relationships between transcription factors and their target genes. The boom in computational biology and various biotechnologies has made inferring GRNs from multi-omics data a hot topic. However, when networks are constructed from gene expression data, they often suffer from false-positive problem due to the transitive effects of correlation. The presence of spurious noise edges obscures the real gene interactions, which makes downstream analyses, such as detecting gene function modules and predicting disease-related genes, difficult and inefficient. Therefore, there is an urgent and compelling need to develop network denoising methods to improve the accuracy of GRN inference.

**Results:**

In this study, we proposed a novel network denoising method named REverse Network Diffusion On Random walks (RENDOR). RENDOR is designed to enhance the accuracy of GRNs afflicted by indirect effects. RENDOR takes noisy networks as input, models higher-order indirect interactions between genes by transitive closure, eliminates false-positive effects using the inverse network diffusion method, and produces refined networks as output. We conducted a comparative assessment of GRN inference accuracy before and after denoising on simulated networks and real GRNs. Our results emphasized that the network derived from RENDOR more accurately and effectively captures gene interactions. This study demonstrates the significance of removing network indirect noise and highlights the effectiveness of the proposed method in enhancing the signal-to-noise ratio of noisy networks.

**Availability and implementation:**

The R package RENDOR is provided at https://github.com/Wu-Lab/RENDOR and other source code and data are available at https://github.com/Wu-Lab/RENDOR-reproduce

## 1 Introduction

Gene regulatory networks (GRNs) are essential components of the cellular machinery that govern gene expression and control various biological processes. GRNs are composed of complex interactions among genes, transcription factors (TFs), and various regulatory elements (Li *et al.* 2024, Yuan and Duren 2024). The rapid growth of computational biology and biotechnology has made inferring GRNs from multi-omics data a prominent research area (Badia-i-Mompel *et al.* 2023, Li *et al.* 2023). Deciphering GRNs is fundamental for understanding how genes are controlled and coordinated, which in turn impacts various cellular processes and contributes to unraveling the molecular mechanisms underlying development and diseases (Conard *et al.* 2021, Kloesch *et al.* 2022).

However, when networks are constructed from data through computational inference methods (e.g. calculating pairwise correlations), they are prone to suffer from false-positive problem due to the transitive effects (Feizi *et al.* 2013, Xiao *et al.* 2022). This can result in the observed network being affected by indirect noise from two perspectives. First, consider the scenario where gene A regulates gene B, and gene B regulates gene C. In this case, a strong correlation between the expression of genes A and C can also be observed, even though there is no direct connection between them (Barzel and Barabási 2013, Feizi *et al.* 2013). Second, the transitive effects can also lead to overestimating the weights of edges linked by multiple indirect network paths. These spurious indirect effects can perturb the true underlying network structure, complicating the analyses of gene interaction patterns. Therefore, it is crucial to decipher the direct relationships between genes from observed networks containing both direct influences (true signals) and indirect influences (noises).

To address the challenge of indirect noises in GRN inference, methodologies typically fall into two main categories. The first involves inferring direct regulatory networks from gene expression data. For example, wpLogicNet (Malekpour *et al.* 2023) infers directed GRN structures and logic gates among genes by using a Bayesian mixture model to estimate target gene profiles. CMI2NI (Zhang *et al.* 2015) and CN (Aghdam *et al.* 2015) utilize Conditional Mutual Information (CMI) to compute causal gene associations. However, CMI is known for its tendency to underestimate, potentially leading to false negatives (Zhao *et al.* 2021). The Partial Mutual Information method was also proposed to infer the partial independence of variables (Zhao *et al.* 2016). Additionally, RSNET (Jiang and Zhang 2022) leverages Mutual Information and recursive optimization for network redundancy reduction. These information theory-based methods perform effectively with discrete data, but their efficacy is reduced with continuous data due to the requirement for a larger dataset, posing a limitation to their applicability.

Alternatively, the second strategy involves denoising an inferred GRN to improve its accuracy. Notably, Network Deconvolution (ND) (Feizi *et al.* 2013) deduces direct dependencies from an observed network using eigenvalue reweighting techniques. Despite its effectiveness, ND lacks robust physical interpretation, as it represents higher-order indirect influences merely by raising the weight matrix A to $A^{k}$, falling short in providing the underlying network dynamics. In a similar vein, Baruch *et al.* formulated Silencer (Barzel and Barabási 2013) to eliminate indirect noises of correlation networks, which treats the observed correlation perturbations as the cumulative outcome of local perturbations. This model, however, is constrained to scenarios where the input matrix is a correlation matrix. In addition, Network Enhancement (NE) (Wang *et al.* 2018) employs a diffusion-based mechanism to denoise biological networks by enhancing the signal intensity through a nonlinear operator. NE defines strong and well-organized edges as network signals and then enhances their weights, which may not hold true across diverse GRNs. Furthermore, NSRGRN refines GRNs by integrating topological properties and edge importance measures (Liu *et al.* 2023). Graph-MRcML recovers direct causal network using a graph deconvolution algorithm (Lin *et al.* 2023). Classical methods like partial correlation (Kim 2015) also provide alternative strategies to remove indirect influences, yet they typically rely on a linear assumption, which may not hold in cases of nonlinear variable interactions and are generally limited to low-order interactions.

**Figure 1.**
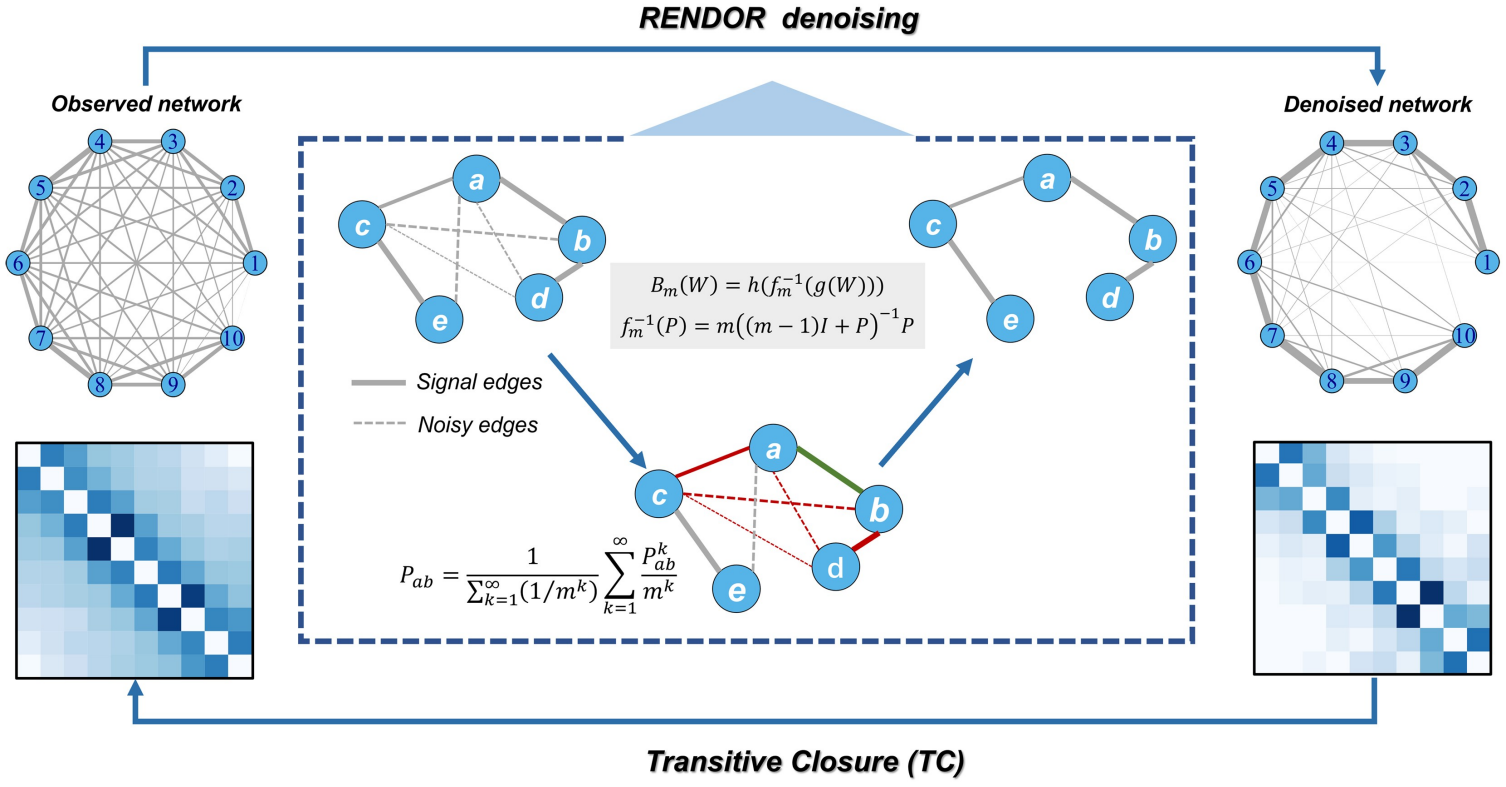
The framework of RENDOR. RENDOR takes a noisy network as input, which is often affected by indirect effects, and outputs a denoised network containing only direct effects. The core of the RENDOR method lies in employing a reverse network diffusion approach based on random walks.

In this study, we proposed a novel network denoising method, named REverse Network Diffusion On Random walks (RENDOR), based on our previous work (Yu *et al.* 2023). RENDOR formulates a network diffusion model under the graph-theory framework to capture indirect noises and attempts to remove these noises by applying reverse network diffusion (Fig. 1). RENDOR excels in modeling high-order indirect influences, since it normalizes the product of edge weights by the degree of the nodes in the path, thereby diminishing the significance of paths with higher intermediate node degrees (Yu *et al.* 2023). The underlying assumption of RENDOR is that the observed noisy network can be conceptualized as an outcome of diffusion from an underlying true network. Consequently, we can use the inverse diffusion to denoise GRNs to improve their inference accuracy accordingly. The rationale for modeling indirect effects as information diffusion lies in the consideration that indirect influences can be deconstructed into composite outcomes resulting from second-order, third-order, and higher-order effects. Global network diffusion provides a better way to account for the influences of different network orders.

## 2 Materials and methods

As a network denoising method, RENDOR is designed to adjust edge weights for improving the signal-to-noise ratio (SNR) of the input network. The network outputted by RENDOR is a weighted complete graph, meaning that edges not present in the original network (with an initial weight of 0) may also acquire a small weight after denoising. Its primary goal is to amplify the weights of direct relationships between variables, while diminishing those of indirect noisy edges, thus enhancing the accuracy of network inference.

We first present an overview of RENDOR in Fig. 1. It takes a noisy network afflicted by indirect effects as input, models higher-order indirect interactions between nodes by transitive closure, eliminates false-positive edges through inverse network diffusion, and ultimately yields the refined network as output.

Specifically, RENDOR decomposes the noisy observed network $G_{\mathrm{obs}}\;$into the sum of a direct network $G_{\mathrm{dir}}\;$and an indirect network $G_{\mathrm{indir}}$. It leverages a network diffusion approach, named Network Refinement (NR), to model indirect influences (Yu *et al.* 2023):

$$G_{\mathrm{obs}}=G_{\mathrm{dir}}+G_{\mathrm{indir}}=\mathrm{NR}(G_{\mathrm{dir}})$$

The underlying assumption is that the observed network $G_{\mathrm{obs}}$ can be obtained through NR diffusion on a true network $G_{\mathrm{dir}}$, which encapsulates only direct relationships. The NR diffusion operator is algebraically articulated as a power series of the transition probability matrix associated with $G_{\mathrm{dir}}$, with parameters constrained to guarantee series convergence.

In contrast, for the reconstruction of $G_{\mathrm{dir}}$ from $G_{\mathrm{obs}}$, the inverse diffusion process of NR, termed RENDOR, is applied to eliminate indirect influences, as denoted by:

$$G_{\mathrm{dir}}=\mathrm{RENDOR}(G_{\mathrm{obs}})$$

Next, we will introduce the detailed mathematical formulas and model descriptions of the forward network diffusion method NR, and the reverse network diffusion method RENDOR.

### 2.1 Random walk on graph

Given a weighted undirected graph G=(V,E,W), where V represents the node set of G, E represents the edge set of G, and W captures the weights of edges in E. Random walk on the graph G can be defined as a finite Markov chain with state-space V and transition matrix $P=D^{-1}W$, where D=W1 is the degree matrix of G. That is to say, if the random variable is currently at state (node) i, then its state has a probability of $P_{ij}=W_{ij}/\sum_{j}W_{ij}$ moving to the neighboring state (node) j in the next step. According to the C-K theorem (Kulik and Soulier 2020), the element of k-th power of transitive probability matrix $P_{ij}^{k}$ tells us the probability of moving from node i to node j through a walk of length k.

### 2.2 Reverse network diffusion on random walks

RENDOR takes a noisy network, denoted as $G_{in}\left(V,E,W\right)$, as model input and outputs a denoised network, represented as $G_{\mathrm{out}}(V,E^{\prime },W^{\prime })$. The core of the RENDOR method is the graph transformation operator $B_{m}$, which is a composite graph operator consisting of three mathematical operators: $f_{m}^{-1}$, g, and h. More specifically, the operator $f_{m}^{-1}$ models the inverse process of diffusion, while the operators g and h facilitate the mapping of this inverse diffusion process onto the underlying graph. The subscript m serves as a hyper-parameter that controls the strength of diffusion, and its role will be further elucidated in the subsequent discussion.

#### 2.2.1 Network diffusion on random walks and its inverse process

Denote P as the set of transition matrices and W as the set of (weighted) adjacency matrices of undirected graphs, where P∈P gives the representation of a random walk on a graph, and W∈W gives the representation of a graph. The diffusion process $f_{m}$ transforms a transition matrix P to another by adding the probability of all paths of different length k joining two nodes, with a smaller weight coefficient $1/m^{k}$ for a longer path of length k (Yu *et al.* 2023):

$$f_{m}:\;P\to P$$

$$f_{m}\left(P\right)=\frac{1}{\sum_{k=1}^{\infty }\left(1/m^{k}\right)}\left(\frac{P}{m}+\frac{P^{2}}{m^{2}}+\frac{P^{3}}{m^{3}}+\cdots \right)$$

(1) =1∑k=1∞1/mk∑k=1∞Pkmk 

(2) =m−1PmI-P-1 

where $\sum_{k}\left(1/m^{k}\right)$ is a normalization factor, and m>1 to ensure that the series converges when k approaches infinity.

It is easy to check that the spectral radius ρ (the supremum of the absolute value of the eigenvalues) of P/m satisfies ρ(P/m)<1 when m>1, which promises the convergence of the infinite series: $f_{m}\left(P\right)=(m-1)P{\left(mI-P\right)}^{-1}$.

Now we focus on the inverse process of the diffusion process defined by $f_{m}$. Considering that diffusion will cause indirect effects, we assume that the random walk on the noisy network is generated from the diffusion on a potential network, then we might make use of the inverse process of $f_{m}$ to eliminate the indirect effects (noises). That is to say, we can treat:

(3) Pobs=fm(Pdir)

where $P_{\mathrm{obs}}$ represents the random walk on the observed noisy network, and $P_{\mathrm{dir}}$ represents the random walk on the underlying true network. Then the inverse operator of $f_{m}$ can recover $P_{\mathrm{dir}}$ from $P_{\mathrm{obs}}$ by removing the indirect effect of all lengths of paths between two nodes.

In detail, the inverse diffusion process $f_{m}^{-1}$ is defined as follows:

(4) fm-1P=mm−1I+P-1P

It is easy to verify that the operators $f_{m}$ defined in Equation (2) and $f_{m}^{-1}$ defined in Equation (4) are inverse operators to each other. (See Theorem 1 and its proof in Supplementary Data). Notice that the operator $f_{m}^{-1}$ shares the same properties as the operator $f_{m}$ (Yu *et al.* 2023) (See Theorem 2 and its proof in Supplementary Data). Besides, we need to promise that the operator $f_{m}^{-1}$ also transform a transition matrix to another, that is to say: $f_{m}^{-1}(P)\in P$ for P∈P. Firstly, we can prove that the sum of each row of the matrix $f_{m}^{-1}(P)$ is 1 for P∈P (See Theorem 3 and its proof in Supplementary Data).

We pointed out that the non-negativity of $f_{m}^{-1}\left(P\right)\;$is not always guaranteed, preventing it from being a standard transition matrix. This is because the basic assumption of using the operator $f_{m}^{-1}$ to remove all indirect effects is that the random walk on the noisy input network is (approximately) generated by the diffusion process defined by the operator $f_{m}$. When the truth is far away from this assumption, the inaccuracy could lead to abnormal results. Particularly, removing the indirect effects of a sparse network is challenging, and negative numbers occur when there is no edge between two nodes or when the weight of two nodes is too small to account for the sum of all paths of different lengths joining them. To solve this ill-conditioned problem, which hinders us from ensuring that $f_{m}^{-1}(P)\in P$, we have provided a corrective approach in practical implementation, as detailed in following Section 2.2.4.

#### 2.2.2 Auxiliary operators mapping between graph and random walk

To facilitate the transformation between the graph and the random walk on the graph, we introduce two auxiliary operators, g and h. These two operators enable us to map the diffusion of the random walk defined by the operators $f_{m}$ and $f_{m}^{-1}$ to the diffusion of the graph, as described in our previous work (Yu *et al.* 2023).

Denote D as the diagonal degree matrix of W, then $g\left(W\right)\;$defines a random walk on the graph whose (weighted) adjacency matrix is W:

g: W →P 

(5) gW=D-1W

The operator h has the opposite effect of g, which recovers the underlying graph of the random walk defined by the transition matrix P:

h:P→W 

(6) hP=α·diagπP

where $\pi \left(P\right)=(\pi_{1},\ldots ,\pi_{n})$ is the stationary distribution of transition matrix P such that πP=π. $\mathrm{diag}\left(x\right)$ means the diagonal matrix whose diagonal element is the vector x, and α is a constant that controls the sum of the weight matrix h(P). The operator h multiplies the transition probability $P_{ij}$ by the stationary distribution of node i, which reflects the degree information of the graph.

#### 2.2.3 Composite graph operator for (reverse) network diffusion

The forward diffusion process and backward diffusion process on random walk defined by $f_{m}\;$and $f_{m}^{-1}$ can now be mapped onto the graph, respectively. The composite graph operator $F_{m}$, which is related to $f_{m}$, is what we previously referred to as the NR diffusion method:

$$F_{m}:W\to W$$

(7) FmW=hfmgW

Similarly, we define the composite graph operator $B_{m}$ related to $f_{m}^{-1}$ as:

$$B_{m}:W\to W$$

(8) BmW=hfm-1gW

RENDOR employs the operator $B_{m}$ to modify the edge weights of noisy networks. $B_{m}$ aims at removing the combined indirect effects of all lengths of paths connecting two nodes. Assuming that the noisy network is approximately generated by the network diffusion defined by the operator $F_{m}$, then hopefully we could utilize the operator $B_{m}$ to remove all the indirect effects.

#### 2.2.4 Model implementation

To denoise an observed network $G_{\mathrm{obs}}\;$with adjacency matrix $W_{\mathrm{obs}}\;$using the RENDOR method, we first define a random walk $g\left(W_{\mathrm{obs}}\right)\;$on the graph, then applied inverse diffusion to get $f_{m}^{-1}\left(g\left(W_{\mathrm{obs}}\right)\right)$, and finally recover the denoised graph $h\left(f_{m}^{-1}\left(g\left(W_{\mathrm{obs}}\right)\right)\right)$.

We have mentioned before that the matrix $f_{m}^{-1}(g(W_{\mathrm{obs}}))$ may not always be non-negative, which is a prerequisite for it to be a standard transition matrix. To solve this ill-conditioned issue, we preprocess the input network by modifying it to ${\tilde{W}}_{\mathrm{obs}}=W_{\mathrm{obs}}+\varepsilon_{1}J+\varepsilon_{2}I$ before applying RENDOR. Here, J is a matrix with all elements equal to 1, and I is the identity matrix with diagonal entries of 1. The parameters $\varepsilon_{1}$ and $\varepsilon_{2}$ are introduced to control the extent of modification to the input network. This preprocessing step can be understood as a recovery mechanism, if we assume that the input network is obtained by thresholding a network and removing the diagonal entries. This harmless modification does not destroy the structure of the original network and helps to make $f_{m}^{-1}(g(W_{\mathrm{obs}}))$ non-negative if we take large enough values for the parameters $\varepsilon_{1}$ and $\varepsilon_{2}$. In order not to affect the properties of the input network, we take $\varepsilon_{1}=\varepsilon_{2}=1\;$for the unweighted graph and the minimum non-zero value for the weighted graph.

However, when the input network is weighted, a preprocessing step like ${\tilde{W}}_{\mathrm{obs}}=W_{\mathrm{obs}}+\varepsilon_{1}J+\varepsilon_{2}I$ may not be adequate to guarantee the non-negativity of $f_{m}^{-1}(g({\tilde{W}}_{\mathrm{obs}}))$ due to the complexity of edge weights distribution. Therefore, we take a postprocessing step to the matrix$\;f_{m}^{-1}(g({\tilde{W}}_{\mathrm{obs}}))$ to ensure its non-negativity. Specifically, we adjust each row containing negative numbers in $f_{m}^{-1}(g({\tilde{W}}_{\mathrm{obs}}))$ by subtracting the smallest negative value in that row. Denote $f_{m}^{-1}(g({\tilde{W}}_{\mathrm{obs}}))$ as $P_{\mathrm{dir}}$, this postprocessing step can be formalized as:

P∼dir=Pdir-β11,…βn1T 

where 1 is the column vector with all elements as 1, $\beta_{i}=0$ if the i-th row of $P_{\mathrm{dir}}$ has no negative elements, $\beta_{i}={\text{min}}_{j}\left\{{\left(P_{\mathrm{dir}}\right)}_{ij}\right\}$if the i-th row of $P_{\mathrm{dir}}$ has at least one negative element, j refers to the column index within the i-th row of the matrix $P_{\mathrm{dir}}$.

We present the pseudocode of RENDOR in Table 1 so that the readers can understand our method more clearly.

**Table 1.** The pseudocode for the RENDOR method.

Pseudocode for RENDOR
Input: $W_{\mathrm{obs}}$: weighted adjacency matrix of observed network;
m: diffusion intensity parameter;
$\varepsilon_{1}$, $\varepsilon_{2}$: preprocessing parameters.
Output: $W_{\mathrm{dir}}$: denoised adjacency matrix of direct network.
$1.\;\;{\tilde{W}}_{\mathrm{obs}}=W_{\mathrm{obs}}+\varepsilon_{1}J+\varepsilon_{2}I$
$2.\;\;P_{\mathrm{obs}}=g\left({\tilde{W}}_{\mathrm{obs}}\right)={\left(\mathrm{diag}\left\{{\tilde{W}}_{\mathrm{obs}}1\right\}\right)}^{-1}{\tilde{W}}_{\mathrm{obs}}$
$3.\;\;P_{\mathrm{dir}}=f_{m}^{-1}\left(P_{\mathrm{obs}}\right)=m{\left(\left(m-1\right)I+P_{\mathrm{obs}}\right)}^{-1}P_{\mathrm{obs}}$
4. for i=1,…,n:
if ${\text{min}}_{j}\left\{{\left(P_{\mathrm{dir}}\right)}_{\mathrm{ij}}\right\}\geq 0$: $\beta_{i}=0$
else: $\beta_{i}={\text{min}}_{j}\left\{{\left(P_{\mathrm{dir}}\right)}_{\mathrm{ij}}\right\}$
$5.\;\;{\tilde{P}}_{\mathrm{dir}}=P_{\mathrm{dir}}-{\left(\beta_{1}1,\ldots ,\beta_{n}1\right)}^{T}$
$6.\;\;W_{\mathrm{dir}}=h\left({\tilde{P}}_{\mathrm{dir}}\right)=\mathrm{diag}\left\{\pi \left({\tilde{P}}_{\mathrm{dir}}\right)\right\}{\tilde{P}}_{\mathrm{dir}}$

The computational complexity of RENDOR is $O(n^{3})$, primarily due to the matrix inversion and multiplication steps involved in processing the input network, where n is the number of nodes in the network (Supplementary Data). RENDOR exhibited relatively fast performance, taking approximately 10 seconds to process a network with 1500 nodes, which is considered acceptable (Supplementary Fig. S1).

## 3 Results

We evaluated the denoising performance of RENDOR on both simulated and real GRNs. In each experiment, we examined how applying RENDOR as a denoising step improved the accuracy of network inference. This was validated by comparing the Area Under the Receiver Operating Characteristic Curve (AUROC) and the Area Under the Precision-Recall Curve (AUPR) scores (Supplementary Data).

### 3.1 Comparable methods

We compared the denoising performance of RENDOR with four other state-of-the-art GRN denoising methods: ND (Feizi *et al.* 2013), NE (Wang *et al.* 2018), Silencer (Barzel and Barabási 2013), and inverse correlation matrix (ICM) (Alipanahi and Frey 2013).

#### 3.1.1 Network deconvolution

Given the observed similarity matrix $G_{\mathrm{obs}}$, ND distinguishes direct dependencies $G_{\mathrm{dir}}$ from the following equation:

$$G_{\mathrm{dir}}=G_{\mathrm{obs}}{\left(I+G_{\mathrm{obs}}\right)}^{-1}$$

In practical implementation, ND used matrix eigen-decomposition to derive $G_{\mathrm{dir}}$. Specifically, ND first scaled the observed matrix $G_{\mathrm{obs}}$ to ensure that the spectral radius of $G_{\mathrm{dir}}$ is less than 1, meaning that all eigenvalues fall within the range of −1 to 1. Subsequently, ND performed an eigen decomposition of $G_{\mathrm{obs}}$ as follows:

$$G_{\mathrm{obs}}=U\Sigma_{\mathrm{obs}}U^{-1}=U\mathrm{diag}\left\{\lambda_{1}^{\mathrm{obs}},\;\lambda_{2}^{\mathrm{obs}}\ldots \right\}U^{-1}$$

Here, U represents the matrix of eigenvectors of $G_{\mathrm{obs}}$, and the elements $\lambda_{1}^{\mathrm{obs}}$,$\;\lambda_{2}^{\mathrm{obs}}$, … are the corresponding eigenvalues of $G_{\mathrm{obs}}$.

In the final deconvolution step, the output matrix can be obtained through the following eigenvalue reweighting:

$$\lambda_{i}^{\mathrm{dir}}=\frac{\lambda_{i}^{\mathrm{obs}}}{1+\lambda_{i}^{\mathrm{obs}}}$$

resulting in:

$$G_{\mathrm{dir}}=U\Sigma_{\mathrm{dir}}U^{-1}=U\mathrm{diag}\left\{\lambda_{1}^{\mathrm{dir}},\;\lambda_{2}^{\mathrm{dir}}\ldots \right\}U^{-1}$$

#### 3.1.2 Network enhancement

NE employs a diffusion-based mechanism to denoise biological networks. Specifically, NE takes a weighted matrix W as input and outputs the denoised matrix whose SNR is enhanced. Mathematically, it first encodes the local structures of W as matrix T:

$$P_{ij}=\frac{W_{ij}}{\sum_{k\in N_{i}}W_{i,k}}{Ι}_{\left\{j\in N_{i}\right\}},\;T_{ij}=\sum_{k=1}^{n}\frac{P_{i,k}P_{j,k}}{\sum_{v=1}^{n}P_{v,k}}$$

where $N_{i}$ means the k nearest neighbors of node i, and $I_{\left\{.\right\}}$ is the indicator function. Then it obtains the output matrix by defining an iterative diffusion process on T:

$$W_{t+1}=\alpha T\times W_{t}\times T+(1-\alpha )T$$

#### 3.1.3 Silencer

The Silencer method defines the input correlation matrix C as the global response matrix, encompassing both direct and indirect effects. It calculates the local response matrix S using the following equation, which eliminates the contribution of indirect effects:

$$S=\left(C-I+D\left(\left(C-I\right)C\right)\right)C^{-1}$$

where I represents the identity matrix, and D(X) sets the off-diagonal terms of X to zero.

#### 3.1.4 Inverse correlation matrix

Since the Silencer method essentially relies on a scaled version of the ICM (Alipanahi and Frey 2013), we also employed this well-established method as a baseline approach (referred to as ICM):

$$S=C^{-1}$$

Pij=-SijSiiSjj, i≠j  1,   i=j

When the input C is a correlation matrix, P represents the partial correlation matrix, and S is commonly known as the precision matrix.

As outlined in the Section 1, one can either infer direct GRNs from gene expression data, or denoise existing GRNs. Partial correlation has been widely used in GRN inference to infer direct connections (Guo *et al.* 2017). Network inference methods based on partial correlation can be regarded as applying correlation followed by this ICM network denoising step. Therefore, ICM is capable of refining networks containing indirect effects to further improve their accuracy.

### 3.2 Simulation experiments

We first generated simulated networks containing indirect noise to evaluate the denoising effectiveness of RENDOR. In these datasets, we had ground-truth labels distinguishing noise (indirect edges) from signals (direct edges), allowing us to accurately assess the predictive performance of edge confidence scores.

Specifically, we take the circular graphs, Erdős–Rényi (ER) random graphs, and BA graphs (Barabási and Albert 1999) as the original true networks, and introduce simulated indirect edges to get noisy networks. The method for adding noisy edges is based on the principle that the more paths connecting two nodes and the shorter these paths, the higher the probability of adding an edge between the nodes. Details for generating simulated networks are provided in Supplementary Data. We can control the number of indirect noisy edges added to generate simulated networks with varying levels of noise (Supplementary Figs S2 and S3). Next, we apply network denoising methods to these noisy networks. We compare how well the denoised networks recover the original network and assess their predictive capability regarding noisy/signal edges.

**Figure 2.**
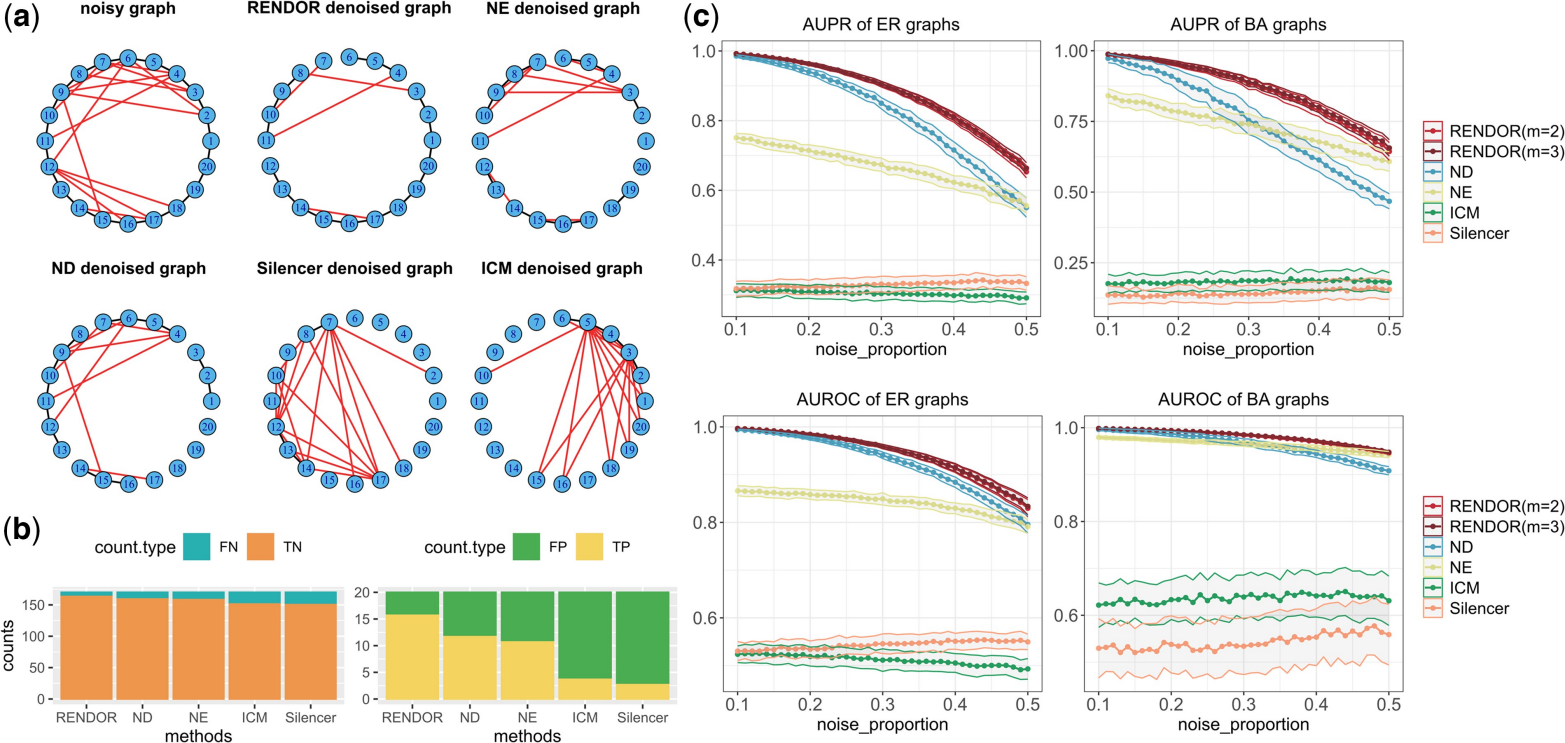
The denoising performance of RENDOR on the simulated networks. (a) Six subfigures present the noisy graph with simulated indirect edges, and the denoised graphs obtained by applying five denoising methods. Noisy edges are marked in red. Ture edges are marked in black. (b) The number of FN, TN, TP, and FP edges of the five denoised networks. (c) The AUROC and AUPR scores (*y*-axis) of applying various denoising methods to the noisy networks under different noise levels (*x*-axis) generated based on the ER graphs and BA graphs.

To visually illustrate the denoising effect of RENDOR, we first created a noisy network by adding 15 indirect edges to a 20-node circular graph with 20 edges (Fig. 2a). After applying five network denoising methods and thresholding the edges to match the original network’s edge count, results in Fig. 2a show that RENDOR more accurately reconstructs the original network structure. Additionally, RENDOR-denoised network contains a higher number of true positive (TP) and true negative (TN) edges, and a lower number of false positive (FP) and false negative (FN) edges (Fig. 2b). This demonstrates the effectiveness of RENDOR in noise elimination.

We further conducted a comparative analysis of five denoising methods on noisy networks generated from the ER graph of node size N=50 and edge-formation probability P=.3, as well as the BA graph of node size N=50 and e=3 (number of edges added each step). To introduce varying difficulty levels in the denoising tasks, we added indirect noisy edges with different intensities. The proportion of noisy edges in the noisy networks ranges from 0.1 to 0.5. Subsequently, we applied five denoising methods to these noisy networks and computed AUPR and AUROC scores. For each proportion of noisy edges, we presented the average score of 100 repeated tests. The results illustrated in Fig. 2c demonstrate that the RENDOR-denoised networks exhibit a higher predictive accuracy, and RENDOR consistently outperforms other methods across different experiment settings. Furthermore, RENDOR displays a smaller variance, underscoring its robustness in handling varying noise levels.

In summary, the simulated experiments designed in this section provide pieces of evidence that RENDOR can effectively and robustly eliminate indirect noise, leading to a more accurate reconstruction of the underlying true network structure.

### 3.3 DREAM benchmark

We then evaluated the denoising performance of RENDOR on the GRNs constructed from the Dialogue on Reverse Engineering Assessment and Methods (DREAM) project (Marbach *et al.* 2012), which serves as a comprehensive platform for assessing the performance of various GRN inference methods (Supplementary Data). Specifically, we obtained GRNs from various network inference algorithms and used them as input for five denoising methods, including RENDOR. We then calculated and compared AUROC and AUPR scores before and after denoising to assess the effectiveness of these methods in enhancing GRN inference.

These GRN inference algorithms tested included correlation-based methods [ARACNE (Margolin *et al.* 2006), Pearson, Spearman], information-theory-based methods [CLR (Faith *et al.* 2007), OIPCQ and OIPCQ2 (Mahmoodi *et al.* 2021)], machine learning approaches [GENIE3 (Huynh-Thu *et al.* 2010), TIGERSS (Haury *et al.* 2012), GRNBoost2 (Moerman *et al.* 2019)], and statistical approaches [Inferelator (Bonneau *et al.* 2006), ANOVA (Küffner *et al.* 2012), wpLogicNet (Malekpour *et al.* 2023)].

#### 3.3.1 RENDOR improves inference accuracy of GRNs

We first utilized the DREAM3 benchmark alongside 13 GRN inference algorithms to assess the denoising efficacy of RENDOR. As illustrated in Supplementary Fig. S4, some network inference methods (like OIPCQ and OIPCQ2) already exhibit excellent performance before denoising, and show further improvement after applying RENDOR. Among the 13 network inference algorithms tested, nearly all GRNs showed improved performance after RENDOR denoising, with the greatest overall improvement compared with other denoising methods.

**Figure 3.**
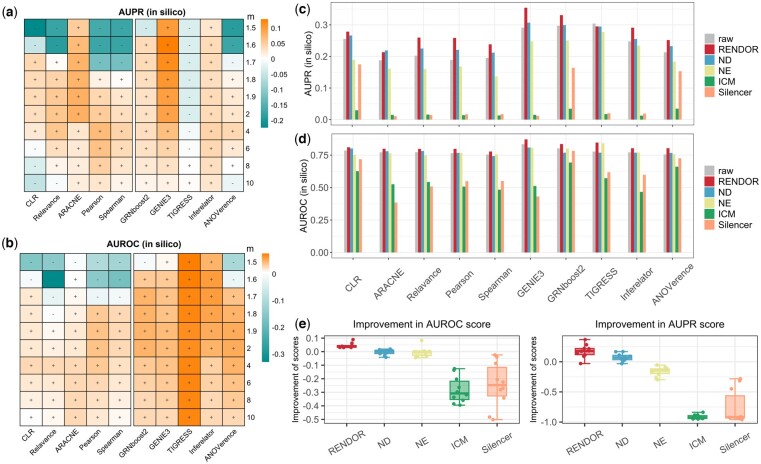
The denoising performance of RENDOR on GRNs that inferred from the DREAM5 dataset. (a) and (b) illustrate the denoising performance of RENDOR on ten GRNs (*x*-axis) for varying values of RENDOR’s parameter m (*y*-axis). The heatmap colors represent the degree of improvement in scores after denoising compared to before denoising. The symbol “+” indicates an enhancement in network accuracy after denoising, whereas “-” denotes a reduction. (c) and (d) present the AUPR and AUROC scores for the ten GRNs before denoising (raw) and after denoising using the five methods. The parameter m of RENDOR was set to 4. (e) Boxplot showing the improvement of AURPC and AUPR scores (*y*-axis) of GRNs derived from various denoising methods (*x*-axis), as compared to the original GRNs. Each dot corresponds to a GRN.

We further tested the denoising performance of RENDOR on DREAM5 *in silico* benchmark. As previously mentioned, RENDOR has a parameter m that modulates the denoising intensity. We now discuss the impact of different values of m on the denoising outcomes. As depicted in Fig. 3a and b, for most values of m, RENDOR exhibits favorable positive denoising effects. However, when m takes on very small values, it leads to a stronger modification of edge weights, which may result in undesirable results. Because if the original network is of high quality, which assumes a lower noise level, excessive denoising is not recommended. Conversely, if the original network is of lower quality, denoising on top of it is theoretically unlikely to yield good results. Therefore, opting for a more conservative m value is strongly recommended. In the subsequent experiments, we present results with m set to 4.

In Fig. 3c and d, we present inference accuracy of 10 GRNs obtained from various network inference algorithms before denoising (raw) and after denoising using five different methods: RENDOR, ND, NE, ICM, and Silencer. It can be summarized that the RENDOR-denoised networks substantially improve the inference accuracy of the input networks due to the diminishing of indirect noise influence, and RENDOR outperforms other methods. The AUROC score has increased by 4.6% overall for RENDOR while reduced by 0.01% for ND. Meanwhile, the AUPR score has increased by 17.0% overall for RENDOR while increased by 7.0% for ND (Fig. 3e). The denoising performance of RENDOR evaluated using F1-score was presented in Supplementary Fig. S5.

Moreover, the denoising performance of both ICM and Silencer was unsatisfactory. This can be attributed to two potential reasons: first, the input matrices were obtained through different methods rather than all derived from correlation; second, the input matrix only gives correlation values between TF and genes, rather than between all genes, so we supplemented the missing values between non-TF genes with zeros to create a square input matrix. It can be concluded that ND and RENDOR are not sensitive to this data preprocessing step, while ICM and Silencer were ineffective at removing indirect effects under this scenario.

#### 3.3.2 Denoising GENIE3-inferred GRN

Furthermore, we observed that the GENIE3 method exhibits the best performance in GRN inference on DREAM5 benchmark, and RENDOR denoising is also more effective based on this high-quality GRN. Therefore, we conducted a detailed analysis of the network structure information of the GRN inferred by GENIE3 and further denoised by RENDOR.

Specifically, for the GRN inferred by GENIE3 on DREAM5 *in silico* dataset and denoised network obtained by applying RENDOR and ND methods on this network, we kept their top 100–1500 edges with the highest weights, and compared the number of correctly inferred edges. Due to the prioritization and elevated ranking of true edges in the network denoised by RENDOR, we observed a significantly higher proportion of TP edges when retaining edges with higher weights. As shown in Table 2, when retaining the top 200 edges with the highest confidence scores, all edges identified by RENDOR correspond to true regulatory relationships. This significantly enhances the network inference accuracy of GENIE3 from 88.5% to 100%. As the number of retained edges increases, the overall inference accuracy decreases. Nevertheless, the GRN denoised by RENDOR consistently demonstrates superior inference performance compared to both the original GRN inferred by GENIE3 and the GRN denoised by ND.

**Table 2.** Comparison of the number of TP edges when different numbers of edges (100–1500) are retained on the original weighted network inferred by GENIE3, and the RENDOR- and ND- denoised networks.

#Kept_edges	GENIE3	GENIE3 + ND	GENIE3 + RENDOR
100	97	99	100
200	177	191	200
300	255	275	299
400	334	354	395
500	405	429	490
1000	711	750	869
1200	794	851	976
1500	901	942	1113

Furthermore, we visualized the RENDOR-denoised GRNs in Supplementary Fig. S6. We observed that they consistently maintain the hub structure in the network without disrupting the scale-free structure present in the original GRN inferred by GENIE3. This indicates that the edge weight adjustments made by RENDOR to the input network are indeed beneficial.

In summary, RENDOR significantly improved the network inference accuracy when compared with various network denoising methods for GRN inference and thus had a better GRN inference performance.

## 4 Discussion

We acknowledged the limitations of denoising when dealing with networks of inferior quality, such as those where inferred edges are nearly indistinguishable from random guesses. In these cases, the effectiveness of even the most advanced denoising methods is constrained, leading to a scenario described as “garbage in, garbage out.” We utilized the *E.coli* dataset from DREAM5 for further illustration. Notably, the accuracy of GRN inferred on this dataset is lower. This suggests that applying denoising methods may not substantially enhance network quality or provide convincingly better results. Nonetheless, REDNOR is still capable of enhancing GRN performance of most network inference methods, with an average improvement level higher than other denoising methods (Supplementary Fig. S7). Furthermore, RENDOR’s weight adjustment for edges with higher confidence can effectively prioritize some actual existing edges, thus enhancing inference accuracy (Supplementary Fig. S8). When denoising networks of inferior quality, we recommend using RENDOR to adjust only the weights of edges with higher confidence scores, while preserving the original weights of edges with lower scores.

Before applying denoising methods, it is necessary to verify the appropriateness of the denoising scenario. Misapplication of denoising methods in inappropriate contexts can lead to suboptimal or even adverse denoising effects. In the context of denoising GRN, as shown in the Supplementary Fig. S9, using other denoising methods designed for better community detection can dramatically decrease the accuracy of GRN inference. The inferior performance of these denoising methods is because they enhance connections within nodes that have self-organizing properties, thus making the network’s triangular structures denser, which is contrary to the goal of removing indirect influences (breaking down triangular structures).

Additionally, the preprocessing and postprocessing steps of RENDOR are heuristic, aimed at ensuring feasible solutions. Their impact on the denoising effects is not fully understood, warranting further research to explore potentially better approaches. Furthermore, the selection of algorithm hyperparameters is empirical. A more comprehensive evaluation and finer selection methods might be necessary.

## 5 Conclusion

In conclusion, this study introduced RENDOR, a novel denoising approach for improving the accuracy of network inference. This method is designed to handle noisy networks affected by indirect effects. It effectively models higher-order indirect interactions between nodes through network diffusion, employs reverse network diffusion to eliminate indirect effects, and outputs refined networks containing only direct signal edges.

Through comprehensive evaluations on both simulated noisy networks and real GRNs, we demonstrated that RENDOR consistently outperforms alternative denoising methods for GRN inference, enhancing the inference accuracy by effectively mitigating the impact of indirect noise. Furthermore, our experiments showcased RENDOR’s robustness across various noise levels, reinforcing its applicability in diverse biological contexts. RENDOR offers a valuable contribution to the field of network inference and provides researchers with a powerful tool for uncovering more accurate and reliable biological network structures.

## Supplementary Material

btae435_Supplementary_Data
