# Supplementary material for: Reverse network diffusion to remove indirect noise for better inference of gene regulatory networks
Source: Bioinformatics. 2024 Jul 4;40(7):btae435. doi: 10.1093/bioinformatics/btae435 (PMC11236096; doi:10.1093/bioinformatics/btae435)
Supplement: btae435_Supplementary_Data [file btae435_supplementary_data.pdf]

Supplementary Materials

Reverse network diffusion to remove indirect noise for better inference of gene regulatory networks

Jiating Yu[1,2,3], Jiacheng Leng[2,3,4], Fan Yuan[2,3], Duanchen Sun[5,*] and Ling-Yun Wu[2,3,*]

[1]School of Mathematics and Statistics, Nanjing University of Information Science & Technology, Nanjing 210044, China,

[2]IAM, MADIS, NCMIS, Academy of Mathematics and Systems Science, Chinese Academy of Sciences, Beijing 100190, China,

[3]School of Mathematical Sciences, University of Chinese Academy of Sciences, Beijing 100049, China,

[4]Zhejiang Lab, Hangzhou 311121, China,

[5]School of Mathematics, Shandong University, Jinan 250100, China.

*To whom correspondence should be addressed.

Contents

1. Theorem Proofs 3
2. Computational Complexity 6
   2.1. Theoretical analysis 6
   2.2. Running time 6
3. Simulated Networks 8
   3.1. Simulation details 8
   3.2. Example simulated networks 8
4. DREAM Benchmark 10
   4.1. DREAM network inference challenge 10
   4.2. Gene expression dataset 10
   4.3. GRN inference methods 11
   4.4. Evaluation metrics 12
   4.5. Denoising performance on the DREAM3 benchmark 14
   4.6. Denoising performance on the DREAM5 *in silico* dataset 15
   4.7. Denoising performance on the DREAM5 *E. coli* dataset 17
5. Misapplication of Denoising Methods 19

1. Theorem Proofs

Notice that the following three statements are equivalent to each other:

(1) The random walk on graph G is reversible.

(2) The graph G is undirected.

(3) The weighted adjacency matrix of graph G is symmetric.

We reiterate the definition of relevant mathematical operators here for the convenience of the readers' quick reference:

- The operator $f_m (m > 1)$ is defined as:

$$f_m\colon\ \boldsymbol{\mathcal{P}} \longrightarrow \boldsymbol{\mathcal{P}}$$

$$f_m(P) = \frac{1}{\sum_{k=1}^{\infty}(1/m^k)} \sum_{k=1}^{\infty} \frac{P^k}{m^k} = (m-1)P(mI-P)^{-1}$$

- The operator f_m^{-1} is defined as:

$$f_m^{-1}\colon\ \boldsymbol{\mathcal{P}} \longrightarrow \boldsymbol{\mathcal{P}}^*$$

$$f_m^{-1}(P) = m\big((m-1)I+P\big)^{-1}P$$

- The operator g is defined as:

$$g\colon\ \boldsymbol{\mathcal{W}} \to \boldsymbol{\mathcal{P}}$$

$$g(W) = D^{-1}W$$

- The operator h is defined as:

$$h\colon\ \boldsymbol{\mathcal{P}} \to \boldsymbol{\mathcal{W}}$$

$$h(P) = \alpha \cdot \mathrm{diag}(\pi(P))P$$

We now present the proof of the theorems stated in the main text.

Theorem 1. **f_m and f_m^{-1} are inverse operators to each other.**

Proof of Theorem 1:

(1) $f_m^{-1}\big(f_m(P)\big) = P$.

Denote $Y = f_m(P) = (m-1)P(mI-P)^{-1}$ for any transition matrix P. Then

$$Y(mI-P) = (m-1)P \qquad \Rightarrow$$

$$mY = (m-1)P + YP = \big((m-1)I + Y\big)P \qquad \Rightarrow$$

$$m\big((m-1)I + Y\big)^{-1} Y = P \qquad \Rightarrow$$

$$f_m^{-1}(Y) = P$$

So, we have $f_m^{-1}\big(f_m(P)\big) = P$.

(2) $f_m\big(f_m^{-1}(P)\big) = P$.

This can be proved in the same way as (1). ■

Theorem 2. **Let P be a transition matrix and $\{X_k\}$ be the random walk defined by P. If the operator f_m^{-1} still represents a transition probability matrix, then it keeps the following properties of P and $\{X_k\}$ unchanged:**

(1) The reversibility of random walk $\{X_k\}$, thus the undirected property of the underlying graph.

(2) The stationary distribution of random walk $\{X_k\}$.

(3) Let G be the graph where $\{X_k\}$ be treated as a random walk, then the degree distribution of G keeps unchanged under operator f_m^{-1}.

Proof of Theorem 2:

(1) If the input Markov chain is reversible, then there exists a reversible distribution π satisfying $\pi_i P_{ij} = \pi_j P_{ji}$ for it. Denote $\Pi = diag(\pi_1, \ldots, \pi_n)$, then we have $\Pi P = P^T \Pi$. Notice that $\Pi I = \Pi P P^{-1} = P^T \Pi P^{-1}$, so $\Pi P^{-1} = (P^T)^{-1}\Pi$. Then

$$(P^{-1})^T \Pi = \Pi P^{-1} \qquad \Rightarrow$$

$$((m-1)P^{-1} + I)^T \Pi = \Pi\big((m-1)P^{-1} + I\big) \qquad \Rightarrow$$

$$\Big(P^{-1}\big((m-1)I + P\big)\Big)^T \Pi = \Pi P^{-1}\big((m-1)I + P\big) \qquad \Rightarrow$$

$$\Pi m\big((m-1)I + P\big)^{-1} P = \Big(m\big((m-1)I + P\big)^{-1} P\Big)^T \Pi$$

So, we have $\Pi f_m^{-1}(P) = \big(f_m^{-1}(P)\big)^T \Pi$.

(2) Let π be the stationary distribution of P such that $\pi P = \pi$. Notice that $\pi I = \pi = \pi P P^{-1} = \pi P^{-1}$, so

$$\pi = \pi P^{-1} \quad \Rightarrow$$

$$m\pi = (m-1)\pi P^{-1} + \pi = \pi P^{-1}\big((m-1)I + P\big) \quad \Rightarrow$$

$$\pi m\big((m-1)I + P\big)^{-1} P = \pi$$

which proves $\pi f_m^{-1}(P) = \pi$, so π is the stationary distribution of $f_m^{-1}(P)$.

(3) We will prove that later. ■

Theorem 3. **The sum of each row of the matrix $f_m^{-1}(P)$ is 1.**

Proof of Theorem 3:

Denote $\mathbf{1}$ as the column vector whose elements are all 1. To prove the sum of each row of the matrix $f_m^{-1}(P)$ is 1, we only need to prove:

$$m\big((m-1)I + P\big)^{-1} P\mathbf{1} = \mathbf{1}$$

Namely, we need to prove:

$$m * \mathbf{1} = ((m-1)I + P)\mathbf{1}$$

Note that the sum of each row of I and P are both 1, so the sum of each row of $(m-1)I + P$ is m. ■

Now we back to the proof of Theorem 2 (3). Because the degree matrix D of the undirected graph can be computed by the stationary distribution of the random walk it defines, thus the degree distribution keeps unchanged if the stationary distribution keeps unchanged under operator f_m^{-1}.

2. Computational Complexity

2.1. Theoretical analysis

The computational complexity of the RENDOR algorithm is primarily determined by the following two operations:

- Inverting the matrix $(m-1)I + P$ requires $O(n^3)$ operations, where n is the number of nodes in the network.
- Multiplying matrices requires $O(n^3)$ operations.

In conclusion, the computational complexity of the RENDOR algorithm is $O(n^3)$, primarily due to the matrix inversion and multiplication steps involved in processing the input network.

2.2. Running time

We tested the running time of RENDOR on a series of randomly simulated networks, the testing process involved following steps:

(1) Generate random networks: Networks of varying sizes were generated, with node counts set to $n =$ 10, 30, 50, 80, 100, 150, 200, 250, 300, 400, 500. For each size, networks were created with different edge densities, specifically d = 10%, 20%, 30%, 50%, 80%, 100%. The Erdős-Rényi model was employed to ensure a comprehensive range of network complexities.

(2) Add random weights: Each network's edges were assigned random weights, simulating realistic weighted network scenarios. This step involved assigning uniformly distributed random weights to all existing edges.

(3) Execute the RENDOR algorithm: RENDOR was applied to each generated network. To ensure robust timing measurements, each experiment was repeated 10 times. The average execution times were measured for each network size and density combination.

The results of the timing tests (Supplementary Fig. 1) revealed several critical insights into the performance of RENDOR. Firstly, the tests confirmed the theoretical

computational complexity of RENDOR, which is $O(n^3)$. This was evidenced by the increase in execution time as the number of nodes grew. However, RENDOR demonstrated relatively fast performance, taking approximately 10 seconds to process a network with 1500 nodes, which is considered acceptable. Secondly, the execution time was observed to be almost independent of edge density of the input network. This indicates that RENDOR efficiently handles networks of varying densities without significant impact on processing time, making it versatile and reliable for diverse network configurations.

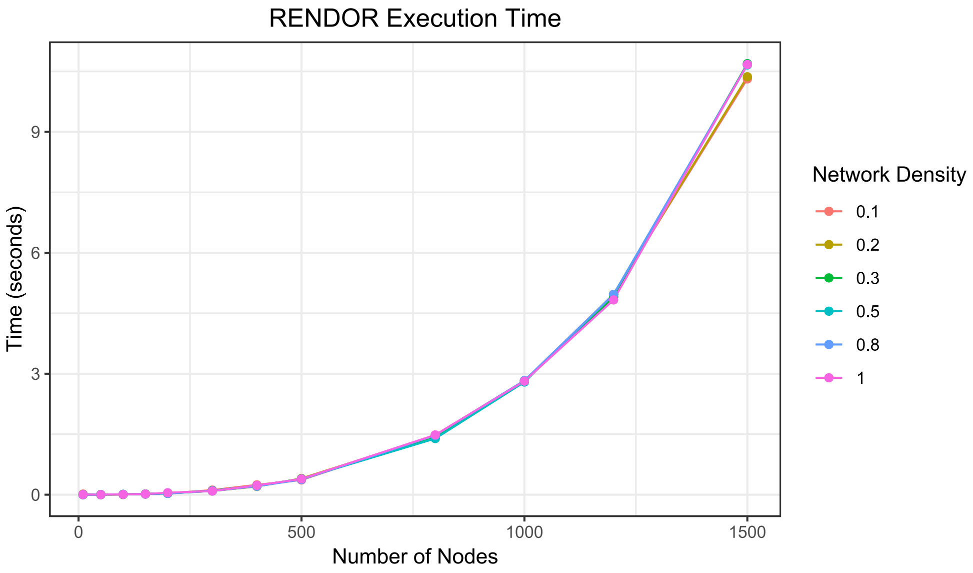

Supplementary Fig. 1. Running time of RENDOR on simulated networks with nodes ranging from 10 to 1500, and network density ranging from 0.1 to 1. The tests were conducted using R programming on a computer equipped with an Intel(R) Xeon(R) CPU E5-2640 v4 @ 2.40GHz.

3. Simulated Networks

3.1. Simulation details

We generated simulated noisy networks by introducing indirect noises into a given graph G with adjacency matrix A. Since we assumed that the observed noisy network G_{obs} can be obtained through NR diffusion of a true network G_{dir} containing only direct relationships, we can add simulated indirect noise to A based on the weighed matrix $W = \mathrm{NR}(A)$. Specifically, we retain the top n edges with the highest weights in the weight matrix W, where n is greater than the number of edges in the original network G. The larger the n, the higher the noise intensity in the simulated network.

After the noise is added, we ensure that the resulting matrix is symmetrical, reflecting the typical characteristic of an undirected network where if node i is connected to node j, then j is also connected to i. Finally, the matrix is converted into a binary format, where each element is either 0 or 1, indicative of the absence or presence of a connection or link.

3.2. Example simulated networks

Here, we show some examples of simulated noisy networks generated based on BA/ER graph under various noise levels (Supplementary Fig. 2, Supplementary Fig. 3).

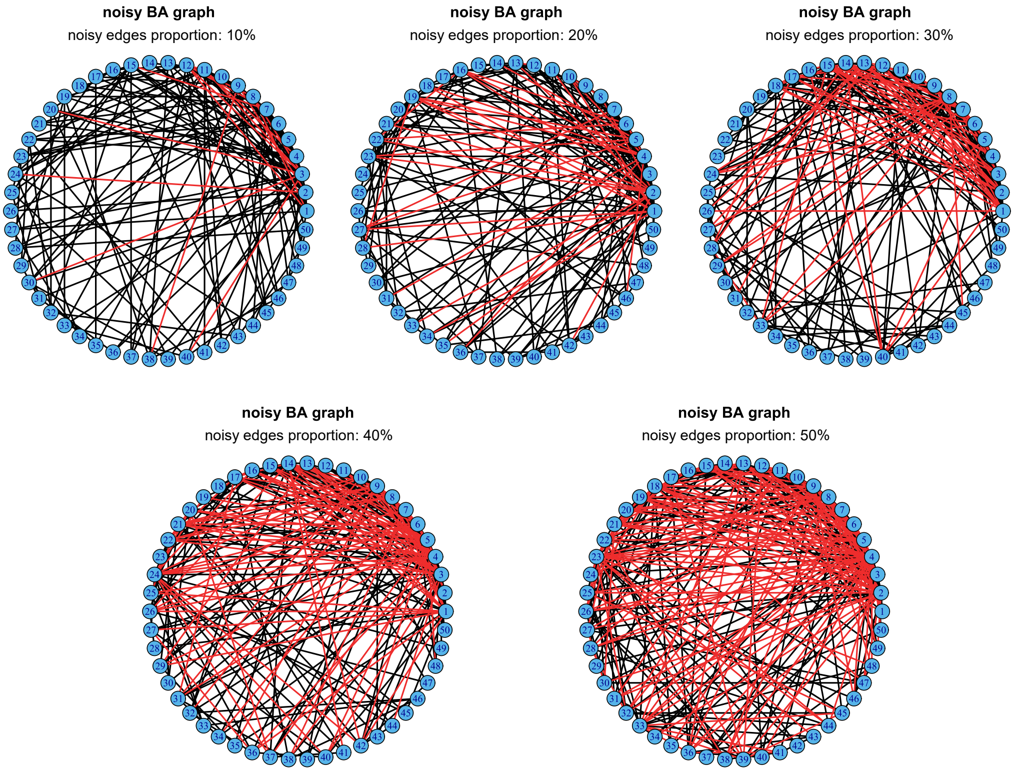

Supplementary Fig. 2. Simulated noisy networks generated based on BA graph under various noise levels.

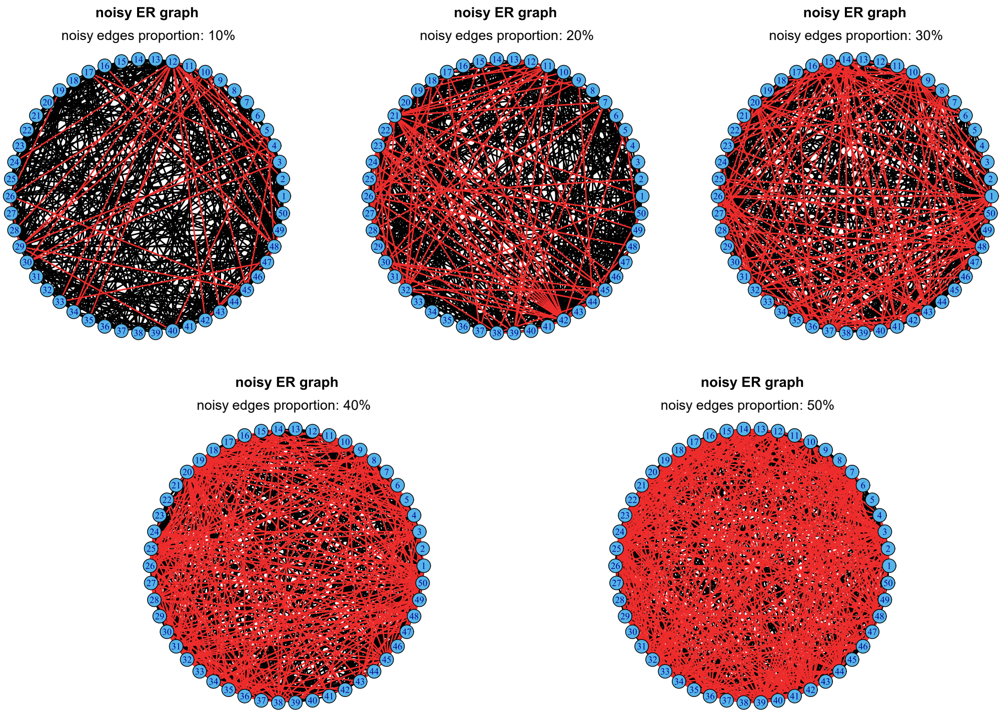

Supplementary Fig. 3. Simulated noisy networks generated based on ER graph under various noise levels.

4. DREAM Benchmark

4.1. DREAM network inference challenge

The Dialogue on Reverse Engineering Assessment and Methods (DREAM) project aims at providing an organized benchmarking platform for accessing the performance of different network inference methods. Participants can run their algorithms to reconstruct Gene Regulatory Networks (GRNs) from gene expression datasets, and evaluated the effectiveness of their algorithms through standardized performance metrics. In this study, we evaluated the GRN inference performance on the DREAM3 benchmark (https://gnw.sourceforge.net/dreamchallenge.html#dream3challenge) and the DREAM5 benchmark (https://dreamchallenges.org/dream-5-network-inference-challenge/).

4.2. Gene expression dataset

We analyzed the 10-node *in silico* dataset provided by the DREAM3 project to evaluate the accuracy of GRNs inferred from these data. This dataset features simulated gene expression data derived from a known regulatory network of 10 genes. The primary goal is to reconstruct the GRN from these expression data, which allows for a robust evaluation of various network inference methods.

We also analyzed two microarray datasets (bulk gene expression data) provided by the DREAM5 project: the *in silico* dataset and the *E. coli* dataset, to evaluate the accuracy of gene regulatory networks inferred from these data. DREAM5 additionally supplied the corresponding gold standard GRN structures for both datasets, serving as ground truths. The gene expression matrix of the *E. coli* dataset was compiled from the Gene Expression Omnibus (GEO) database, and includes 4511 anonymous genes (of which 334 are transcription factors) recorded across 805 chip measurements. The *in silico* dataset presents an artificially synthesized gene expression matrix featuring 1643 anonymous genes (with 195 transcription factors) across 805 chip measurements. In

addition, the gene expression profiles for the *in silico* dataset were created using GeneNetWeaver, and is a counterpart to the *E. coli* dataset.

4.3. GRN inference methods

To assess the denoising efficacy of RENDOR, we first utilized the gene expression dataset from the DREAM3 platform alongside 13 GRN inference algorithms: CLR, Relevance, ARACNE, Pearson, Spearman, GENIE3, GRNboost2, TIGRESS, Inferelator, ANOVA, wpLogicNet, OIPCQ, and OIPCQ2. These algorithms were categorized into correlation-based methods (ARACNE, Pearson, Spearman), information-theory-based methods (CLR, Relevance, OIPCQ, OIPCQ2), machine learning approaches (GENIE3, TIGRESS, GRNboost2), and statistical approaches (Inferelator, ANOVA, wpLogicNet).

The implementations of these methods were as follows:

- CLR and ARACNE were implemented using R package "minet"
- GENIE3 was implemented using R package "GENIE3"
- GRNboost2 was implemented using python package "arboreto"
- TIGRESS was implemented using R package "tigress"
- Inferelator was implemented using python package "inferelator"
- ANOVAance was implemented using MATLAB, follow the instructions in https://github.com/andreazorro/Anova
- OIPCQ and OIPCQ2 were implemented using MATLAB, follow the instructions in https://github.com/haammim/OIPCQ-and-OIPCQ2/tree/master
- wpLogicNet was implemented using R, follow the instructions in https: //github.com/CompBioIPM/wpLogicNet

Additionally, we also tested the first ten GRN inference methods on the DREAM5 benchmark. The GRNs were either downloaded directly from the DREAM5 website or generated by running the source code of the network inference methods on the DREAM5 gene expression datasets. Specifically, the first nine methods allow for the

direct download of GRNs from the DREAM5 Challenge (https://dreamchallenges.org/dream-5-network-inference-challenge/) , with availability also in the ND code repository (https://www.nature.com/articles/nbt.2635#Sec12). For the GRNBoost2 method, we utilized the ‘arboreto’ Python package, following the implementation guidelines provided in the referenced study to generate GRN (https://arboreto.readthedocs.io/en/latest/algorithms.html).

4.4. Evaluation metrics

Given that the gene expression datasets provided by the DREAM platform come with corresponding true gene regulatory network structures, upon obtaining a GRN, we can directly compute two classic metrics to assess the accuracy of network inference: the area under the precision-recall curve (AUPR) and the area under the receiver operating characteristic curve (AUROC).

It should be noted that the DREAM evaluation framework does not directly utilize AUPR and AUROC scores. Instead, by simulating a null distribution for 25,000 random networks, these metrics are transformed into their respective p-values, yielding p_{AUROC} and p_{AUPR}. The final score used for ranking is defined as the negative mean of the log-transformed p-values of the two metrics. The DREAM platform provides a standard codebase for the evaluation of network inference algorithms, available at https://static-content.springer.com/esm/art%3A10.1038%2Fnmeth.2016/MediaObjects/41592_2012_BFnmeth2016_MOESM584_ESM.zip.

In this study, we opted to use AUPR and AUROC directly as our evaluation metrics, without utilizing the p-value computation method provided by DREAM. This is because we observed that under DREAM’s metrics, the denoised GRN could sometimes exhibit excessively high significance, leading to an infinite final score.

The methods for calculating AUPR and AUROC scores are as follows:

- **AUPR (Area Under the Precision-Recall Curve)**

AUPR refers to the Area Under the Precision-Recall curve, which is often used to measure the performance of an algorithm for a binary classification problem. The

precision and recall are defined as follows:

$$\text{Precision} = \frac{\text{TP}}{\text{TP} + \text{FP}}$$

$$\text{Recall} = \frac{\text{TP}}{\text{TP} + \text{FN}}$$

where TP represents true positive, which is the number of samples correctly classified as presence; and FP represents false positive, which is the number of samples wrongly classified as presence; and FN represents false negative, which is the number of samples wrongly classified as an absence.

A higher value of AUPR indicates better performance.

- **AUROC (Area Under the Receiver Operating Characteristic Curve)**

AUROC refers to the Area Under Receiver Operating Characteristic Curve, which is also used to measure the performance of an algorithm for a binary classification problem. ROC curve takes the FPR (False Positive Rate) as the x-axis and the TPR (True Positive Rate) as the y-axis. The TPR and FPR are defined as follows:

$$TPR = \frac{TP}{P} = \frac{TP}{TP + FN}$$

$$FPR = \frac{FP}{N} = \frac{FP}{FP + TN}$$

where TP, FP, FN have the same meaning as in AUPR. Besides, TN represents true negative, which is the number of samples correctly classified as absence; P represents the number of real positive cases in the data, and N represents the number of real positive cases in the data.

A higher value of AUROC indicates better performance.

Additionally, we also tested the F-measure scores on some of these datasets to evaluate the performance of the GRN inference.

- **F-measure**

The F-measure (also known as F1-score) is the harmonic mean of precision and recall scores:

$$\text{F1} = 2 \cdot \frac{\text{Precision} \cdot \text{Recall}}{\text{Precision} + \text{Recall}}$$

4.5. Denoising performance on the DREAM3 benchmark

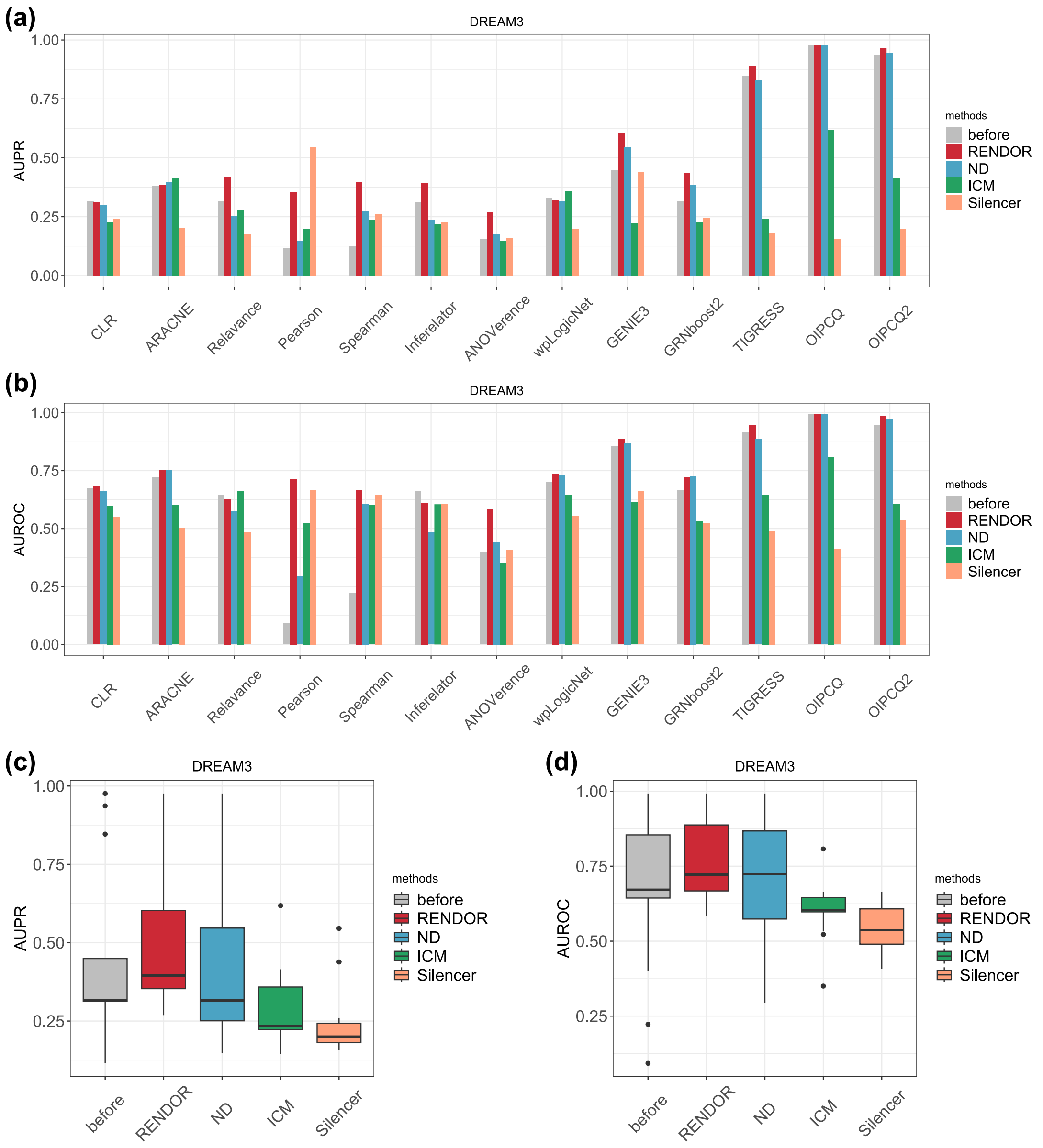

Supplementary Fig. 4. The **(a)** AUPR and **(b)** AUROC scores of four network denoising methods (RENDOR, ND, ICM, Silencer) on 13 GRNs inferred from the DREAM3 dataset. **(c)(d)**: Boxplots showing the inference performance of the GRNs obtained by the network inference algorithms themselves (before) compared to the GRNs obtained through four network denoising methods.

4.6. Denoising performance on the DREAM5 *in silico* dataset

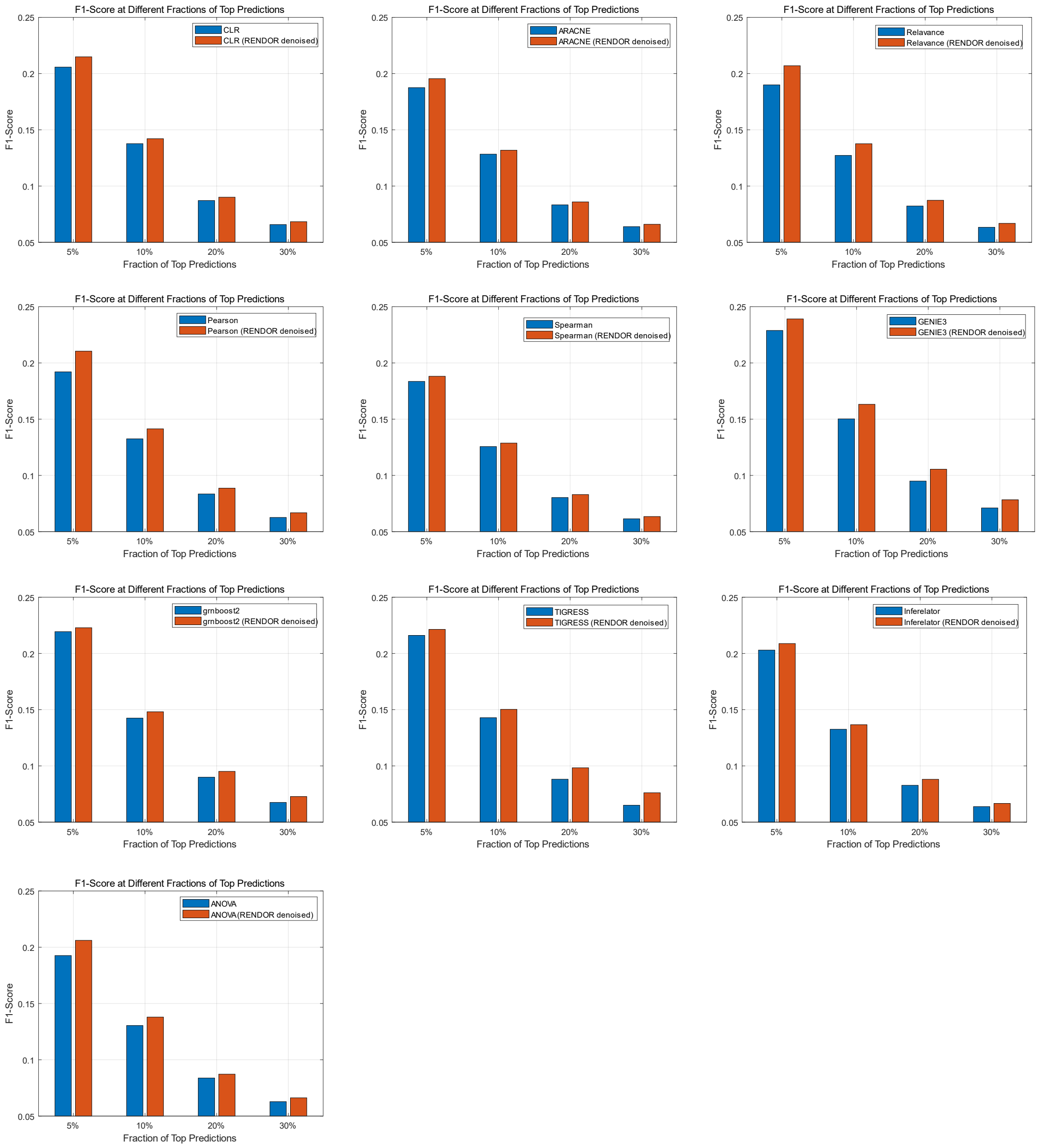

Supplementary Fig. 5. The comparison of improvements in F-measure scores before and after RENDOR denoising for GRNs inferred by 10 network inference methods from the DREAM5 *in silico* dataset. The comparison was made by retaining the top 5%, 10%, 20%, and 30% of the predictions.

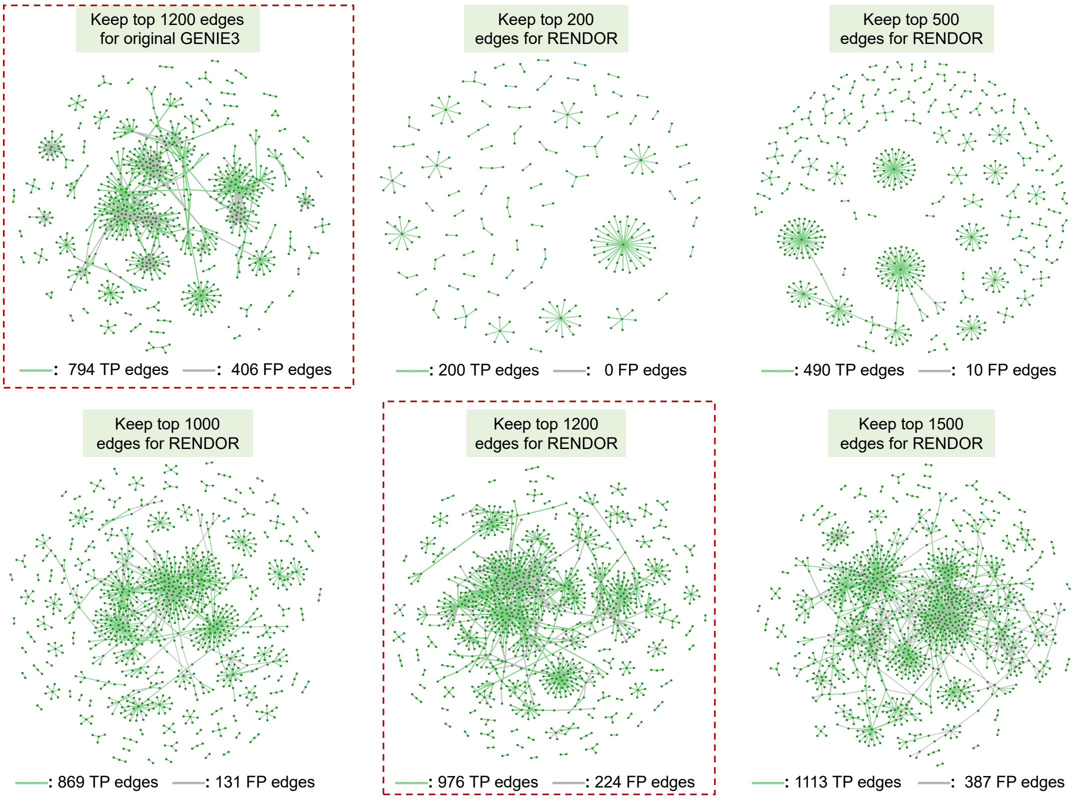

Supplementary Fig. 6. Presentation of the number of TP edges when different numbers of edges are retained on the RENDOR denoised network. For the weighted network obtained by applying RENDOR on the original weighted network inferred by GENIE3, we keep its top 200, 500, 1000, 1200, 1500 highest scoring edges and get 200, 490, 869, 976 and 1113 correctly inferred edges respectively. Due to the prioritization and higher ranking of true edges in the net-work denoised by RENDOR, the proportion of TP edges within the top 1200 retained edges of the RENDOR-denoised network is higher compared to the proportion of TP edges in the top 1200 edges of the original network inferred by GENIE3.

4.7. Denoising performance on the DREAM5 *E. coli* dataset

In Figure 3 of the main text, we demonstrate the denoising effect on the DREAM5 *in silico* dataset. In this section, we extend our analysis to include the denoising performance on another DREAM5 dataset, the *E. coli* (*Escherichia coli*) dataset, which was compiled from the GEO database, for conduct further benchmark evaluations.

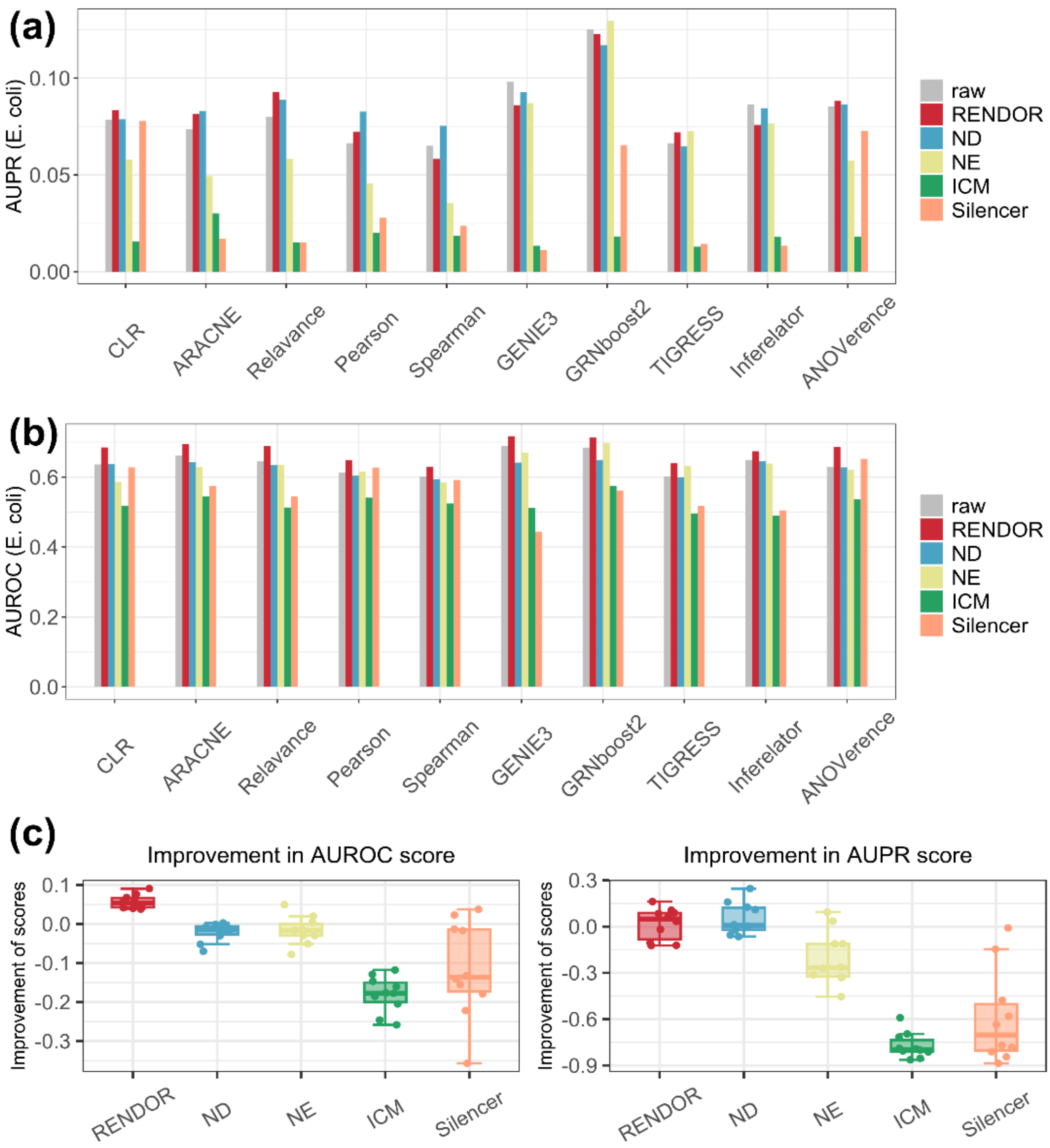

Supplementary Fig. 7. The performance of five denoising methods on the DREAM5 *E. coli* (*Escherichia coli*) dataset. The labels, legends, colors, and other information are consistent with those in Figure 3 of the main text.

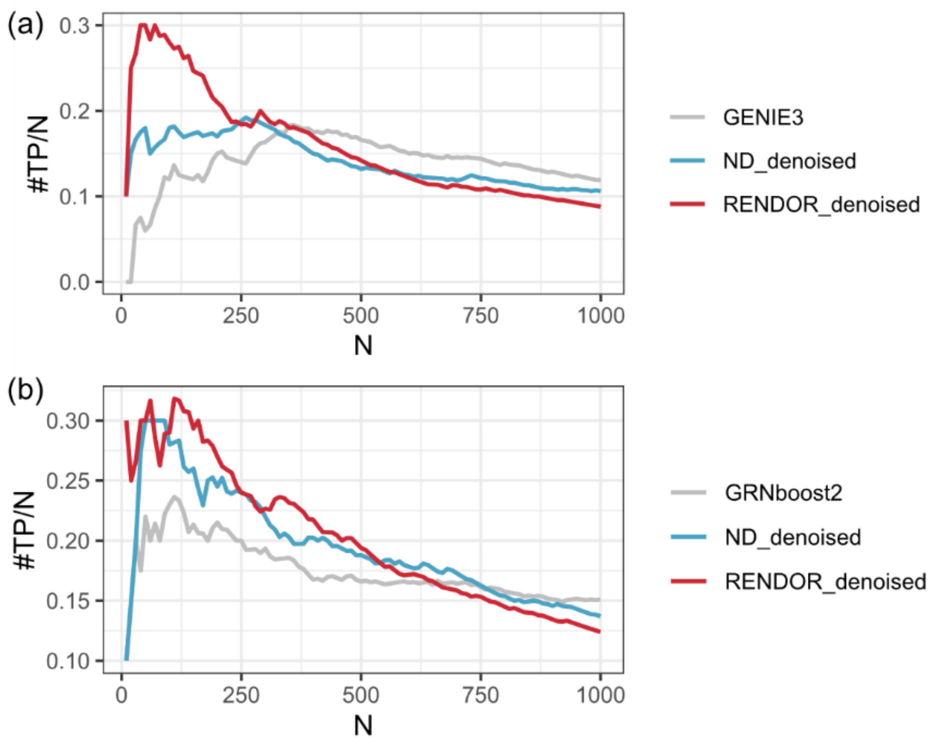

Supplementary Fig. 8. We retained the top N (x-axis) edges in the original GRN inferred by GENIE3 **(a)** and GRNboost2 **(b)**, the RENDOR-denoised GRN, and the ND-denoised GRN, and calculated the proportion of true positive (TP) edges among them (y-axis).

5. Misapplication of Denoising Methods

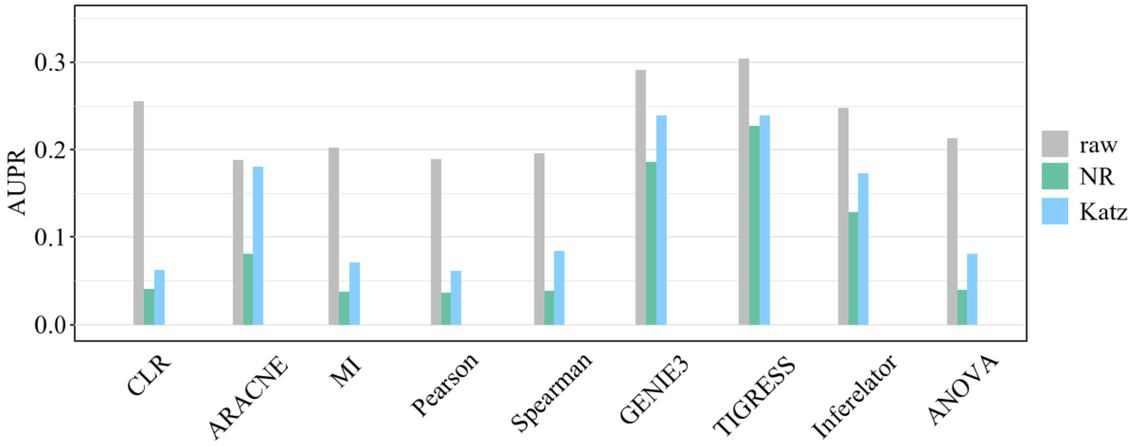

Supplementary Fig. 9. The AUPR scores (y-axis) for the GRNs (x-axis) before denoising (raw) and after denoising using the two methods (NR, Katz). Misapplication of denoising methods designed for better community detection in GRN contexts can dramatically decrease the accuracy of GRN inference.
